# Trio-colored appraisal of an eco-conscious method for the determination of bisphenol A in drinking water bottles and pharmaceutical eye-drop solutions

**DOI:** 10.1186/s13065-025-01713-w

**Published:** 2026-01-23

**Authors:** Farah S. Elbitar, Lobna A. Hussein, Noha M. El Zahar, Hend Z. Yamani, Fotouh R. Mansour

**Affiliations:** 1 Medicinal Chemistry Department, Faculty of Pharmacy, King Salman International University (KSIU), Ras Sudr, South Sinai, 46612 Egypt; 2 Pharmaceutical Analytical Chemistry Department, Faculty of Pharmacy, Ain Shams University, Organization of African Unity Street, Abbassia, 11566 Cairo Egypt; 3 Pharmaceutical Analytical Chemistry Department, Faculty of Pharmacy, Tanta University, Tanta, 31111 Egypt

**Keywords:** Bisphenol A, Liquid-liquid microextraction, Green analytical chemistry, Endocrine disruptors, Water analysis

## Abstract

Bisphenol A (BPA) is a hazardous endocrine-disrupting compound which is frequently detected in water sources due to its extensive application in plastic manufacturing. This study introduces an environmentally sustainable liquid-liquid microextraction (LLME) approach, coupled with high-performance liquid chromatography with diode-array detection (HPLC/DAD), for the determination of BPA in drinking water bottles and pharmaceutical eye-drop solutions. 1-Decanol was employed as a green extraction solvent, and critical experimental parameters were systematically optimized to achieve maximum extraction efficiency. Furthermore, the mobile phase consisted of ethanol and water (40:60 v/v), both recognized as green solvents, further enhancing the method’s eco-friendly profile. The developed method demonstrated accepted linearity (*r* = 0.9989) within the concentration range of 25–20,000 ng mL^− 1^. The high recovery rates (98.23%–101.73%) and the low relative standard deviations (RSD ≤ 3.0%) confirmed the method’s accuracy and precision. The method’s environmental sustainability was confirmed through comprehensive greenness and performance evaluations using the Analytical Eco-Scale, Modified Green Analytical Procedure Index (MoGAPI), Analytical GREEnness Metric Approach (AGREE), Click Analytical Chemistry Index (CACI) and the Environmental, Performance, and Practicality Index (EPPI)metrics. In addition, the carbon footprint reduction index (CaFRI) score of 78 demonstrated a substantial decrease in environmental burden, while the red analytical performance index (RAPI) score of 75 reflected strong analytical performance suitable for routine laboratory application. The developed method achieved a greenness of 78% (MoGAPI), blueness of 78%, and redness of 75%, collectively indicating full compliance with the white analytical chemistry (WAC) framework. Together, this trio-colored assessment system effectively integrates environmental sustainability with analytical performance, offering a holistic paradigm for method evaluation. These findings position the proposed method as a viable and reliable alternative for BPA analysis in water samples and pharmaceutical solutions packed in plastic containers.

**Supplementary Information:**

The online version contains supplementary material available at 10.1186/s13065-025-01713-w.

## Introduction

 Bisphenol A (BPA) is a synthetic organic compound widely used as a building block in the production of various polymeric materials. It is primarily used in the manufacture of polycarbonate plastics and epoxy resins. This type of plastics is used to produce food and liquid containers (including tableware, microwave ovenware, cookware, and water dispenser reservoirs) as well as non-food items such as toys and pacifiers for protective personal care shields [1]. Based on regulatory evaluations, the European Food Safety Authority (EFSA) has established a tolerable daily intake of 0.2 ng BPA/kg body weight per day, highlighting that even trace amounts of BPA in drinking water can contribute substantially to daily exposure and may pose a health concern [2, 3]. Due to its widespread use, BPA contamination has become a major health concern, as it can migrate into food and drinks, leading to human exposure. Several recent studies have highlighted alterations that could suggest BPA’s impact on brain development [4, 5]. BPA is also classified as an endocrine-disrupting chemical, due to the similarity between BPA and estrogen structure [6]. This chemical similarity induces estrogen like endocrine effects which interfere with hormonal regulation [7]. BPA is also associated with certain types of cancer as breast cancer and prostate cancer [8–10]. As a result for all these causes, it is crucial to find accurate, eco-friendly analytical techniques for its detection and quantification.

Advances across the analytical sciences continue to demonstrate the growing need for reliable, sensitive and sustainable detection strategies for a wide spectrum of chemical contaminants. Whether the target analytes are environmental pollutants, pharmaceuticals [11, 12], pesticides [13], or other biologically active substances, their presence in various matrices can pose significant risks, and the development of analytical methods for their identification has expanded rapidly in recent years [14, 15]. Numerous studies highlight how contaminants regardless of whether they originate from therapeutic agents, industrial chemicals, or natural degradation products may contribute to adverse biological or toxicological outcomes, emphasizing the importance of robust and well-validated monitoring approaches [16, 17].

According to the literature, different analytical methods have been developed for BPA determination, including, gas chromatography-mass spectrometry [18], Raman spectroscopy [19, 20] and high performance liquid chromatography (HPLC) coupled to various detectors such as ultraviolet detector (UV) [21], photo-diode array detector (DAD) [22], fluorescence detector [23], mass spectrometry [24, 25] or electrochemical sensors [26]. However, to ensure accurate detection at very low concentrations, appropriate sample preparation is crucial before instrumental analysis. Traditional methods such as liquid-liquid [27] and solid-phase extractions [23] have been commonly employed. More advanced microextraction techniques, including dispersive liquid-liquid microextraction (DLLME) [28] and solid-phase microextraction [29] have also been reported. These microextraction approaches offer significant advantages by minimizing solvent consumption while achieving high analyte enrichment, making them efficient and environmentally friendly alternatives for BPA analysis.

Building on these advances, recent studies have also explored greener LLME strategies for BPA extraction, particularly those employing deep eutectic solvents (DESs) and ionic liquids (ILs). Thymol-based, pH-responsive DESs prepared with medium-chain carboxylic acids have shown excellent extractive capability for BPA through reversible, stimulus-controlled phase transitions, achieving high sensitivity and recovery in water samples [30]. In addition, a dl‑carnitine-based hydrophobic DES was applied in ultrasound-assisted dispersive liquid-phase microextraction (USA‑DLPME) of BPA in mineral water, attaining nearly complete recoveries (~ 99.9%) [31]. Among IL-based strategies, imidazolium IL-functionalized molecularly imprinted sorbents (ZIF-67@[Bmim][Br]@MIP) combined with HPLC achieved high selectivity and sensitivity in aqueous BPA samples [32]. Collectively, these DES- and IL-based approaches illustrate the potential of green solvents for rapid, efficient, and environmentally friendly BPA extraction, supporting the rationale for employing 1-decanol in the present study.

Even though numerous methods for BPA analysis in different matrices have been reported, combining a green analytical method with an advanced eco-friendly sample preparation technique with comprehensive assessment of all analytical aspects is still needed. Accordingly, this study aims to integrate a green chromatographic method with liquid-liquid microextraction (LLME) to develop a highly practical, quick, and reliable method for BPA determination without any environmental burdens. This approach not only enhances analytical efficiency but also aligns with global sustainability efforts and regulatory requirements for safer chemical analysis.

Several tools have been developed to evaluate the sustainability of analytical methods, each offering a distinct perspective on environmental impact and performance. GAPI provides a stepwise pictogram covering all stages of the analytical workflow [33], while AGREE consolidates the twelve principles of green analytical chemistry into a single quantitative score [34]. The Environmental, Performance, and Practicality Index (EPPI) focuses on evaluating analytical methods by assessing reagent hazards, environmental impact, performance, and practicality [35], while the WAC framework provide a complementary categorization of methods into greenness, analytical performance, and practicality [36]. Building on these concepts, the trio-colored RGB approach integrates these dimensions into a single, unified visual framework that reflects environmental impact, operational feasibility, and analytical quality. Accordingly, this study apply a trio-colored analytical appraisal that quantitatively integrates greenness (environmental impact), blueness (practicality and resource efficiency), and redness (analytical quality), thereby providing a holistic metric for sustainable analytical evaluation.

One important consideration in sustainable method development is the inevitable trade-off between environmental greenness and analytical performance. Greener methods often rely on less hazardous solvents or simplified procedures, which can sometimes lead to compromises in sensitivity, selectivity, or robustness. The assessment presented in the manuscript addresses this issue by evaluating greenness, analytical performance, and practicality/resource efficiency as separate yet complementary dimensions. Considering these aspects side by side makes it possible to identify any imbalance for example, when an improvement in greenness may be accompanied by reduced performance or operational feasibility. This integrated perspective ensures that gains in sustainability do not obscure essential analytical requirements or the practical considerations necessary for routine laboratory application.

## Methodology

### Reagents and solutions

All reagents were of high-purity grade and utilized without further modification. BPA was purchased from Merck (Darmstadt, Germany). HPLC-grade ethanol was obtained from Fisher Scientific (Waltham, MA, USA). 1-Decanol was purchased from Sigma-Aldrich (St. Louis, MO, USA). Ultrapure water was obtained by Omnia pure and ultrapure water systems. NaCl was purchased from Alfa Chemical (Hadayek El-Kobba, Cairo, Egypt). Dasani (The Coca-Cola Company, USA), Nestlé Pure Life (Nestlé Waters, Switzerland), and EAU (El Nour Industry, Egypt) water bottles were obtained from a local market. Farcolin^®^ (Salbutamol sulfate, Pharco Pharmaceuticals, Egypt) and Tobrin^®^ (Tobramycin, EIPICO, Egypt) were purchased from local pharmacies for analysis. Ethanol stock solutions of BPA (1 mg mL^− 1^) were prepared by dissolving 10 mg of BPA in 10 mL ethanol. From this stock, aliquots were taken to prepare a working standard solution of 25 µg mL^− 1^ using water as a diluent.

### Instrumentation and chromatographic conditions

Throughout this study, all chromatographic analyses were performed using an HPLC-ARC system (Waters Corporation, Milford, MA, USA) equipped with a DAD for detection. Chromatographic separation was performed on an XBridge^®^ C18 column (4.6 mm × 50 mm i.d., 3.5 μm particle size). A mobile phase consisting of a mixture of ethanol and water (40:60 v/v) was delivered at a flow rate of 1.00 mL/min. The injection volume was 10 µL, and the DAD was set at 277 nm.

### LLME procedure

**Fig. 1:**
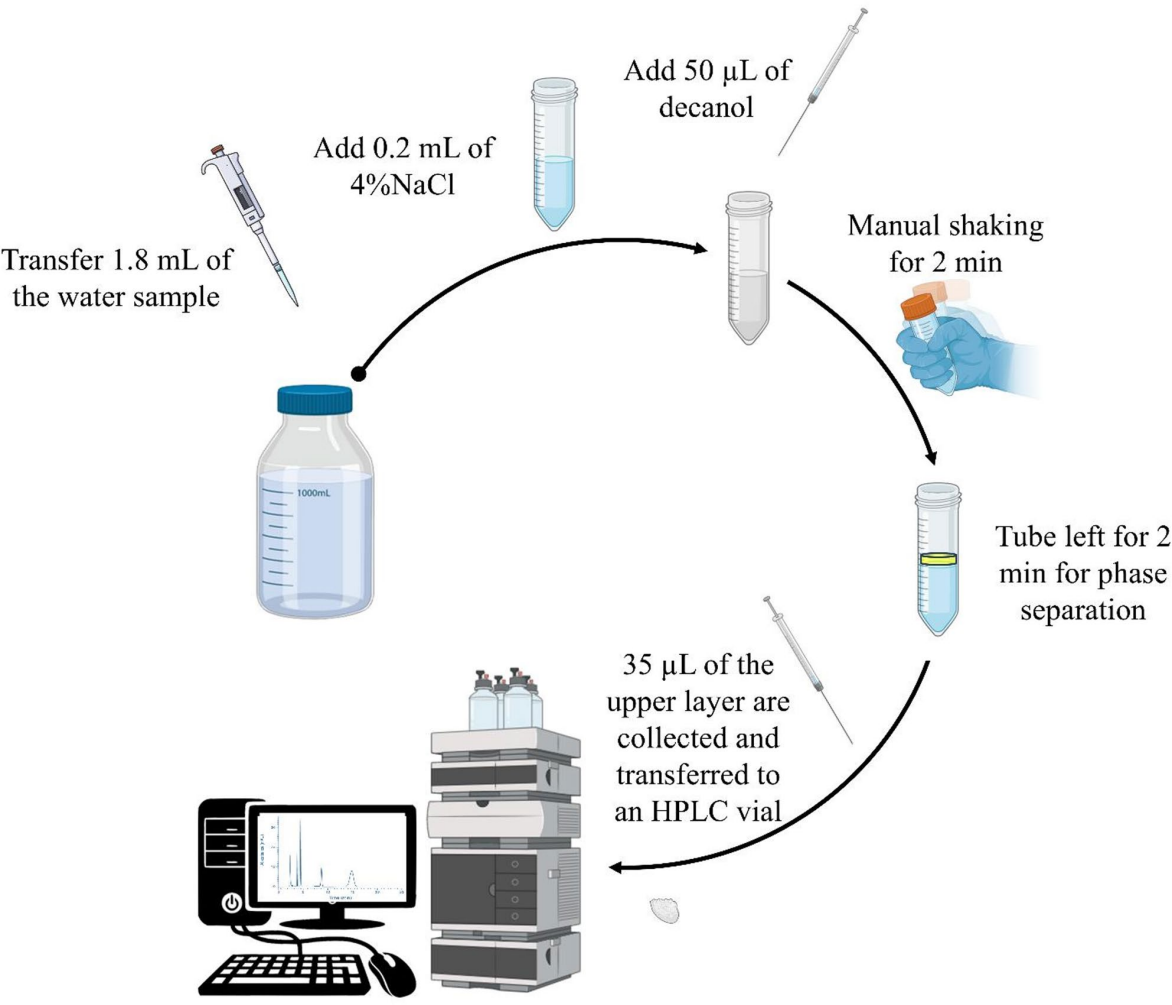
The procedures of BPA determination by LLME HPLC/DAD in drinking water

For the LLME procedure, 1.8 mL of the aqueous BPA solution was transferred into a suitable container. Then, 0.2 mL of 4% (w/v) NaCl solution was mixed. A volume of 50 µL of 1-decanol was added using a chromatographic syringe, and the mixture was manually shaken for 2 min to form a turbid solution. Then it was left for about 2 min to allow the two layers to separate. Approximately 35 µL of the upper layer was carefully collected and transferred into HPLC vials for analysis. The procedures of BPA determination by LLME HPLC/DAD in drinking water is depicted in Fig. 1.

## Results

Chromatographic method development for the separation of BPA was carried out through a structured optimization of the mobile phase composition and flow rate to achieve optimal retention, peak symmetry, and analyte enrichment. The initial mobile phase, comprising acetonitrile and water in a 50:50 (v/v) ratio at a flow rate of 0.5 mL/min, resulted in an unretained peak eluting before one minute. This indicated insufficient interaction between BPA and the stationary phase, rendering the conditions unsuitable for quantitative analysis. Decreasing the acetonitrile content from 50% to 30% resulted in improved retention, but the peak area was inconsistent [21]. Moreover, acetonitrile is considered a less sustainable solvent under green analytical chemistry criteria. To address this, acetonitrile was replaced with ethanol as a greener organic modifier, and the mobile phase was adjusted to a water: ethanol ratio of 70:30 (v/v). Under these conditions, a significant improvement in area and retention repeatability was observed, as evidenced by a pronounced and well-resolved peak, appearing at approximately 14 min. While retention was markedly enhanced, the extended run time necessitated further refinement. By increasing the ethanol content to 40% (i.e., water: ethanol 60:40, v/v), retention time was substantially reduced to 5 min, while maintaining the desired analyte enrichment and peak integrity. Subsequently, increasing the flow rate to 1.0 mL/min further reduced the retention time to 2.8 min, without compromising chromatographic performance. This final set of conditions offered a well-balanced method in terms of efficiency, enrichment, and analysis time, demonstrating its suitability for routine determination of BPA. The corresponding system suitability parameters, including retention time, retention factor, theoretical plate number (N), and tailing factor are summarized in Table S1.

### Optimization of LLME procedure for enhanced BPA enrichment

The LLME procedure was carefully optimized to maximize the enrichment of the target analyte. Key experimental parameters such as extraction solvent volume, pH, mixing time, and salt addition were systematically evaluated in triplicates (*n* = 3) to determine their effects on extraction efficiency. The comprehensive optimization of these parameters ensures the efficiency of the LLME method, making it an attractive alternative for BPA analysis in water samples.

#### Selection of extraction solvent

Originally, LLME used highly hydrophobic and high-density organic solvents (dichloromethane, chloroform, methylene chloride) as an extraction solvent. Over time, the concept of LLME was extended and it became crucial to use smaller volumes and greener solvents than these high-density halogenated organic solvents to reduce their environmental impact. Accordingly non-halogenated, low density solvents (LDS) such as 1-decanol, 1-undecanol & 1-dodecanol were adopted as safer and more environmentally friendly alternatives [37].

Selecting the appropriate extracting solvent is based on how it will provide the ultimate extraction efficiency and also taking into consideration its environmental sustainability. Therefore, 1-decanol was selected due to its classification as a short-chain alcohol, which contributes to lower viscosity and moderate hydrophobicity compared to other LDS. Due to these different chemical properties, it offers a balanced partitioning behavior, making it suitable for a broader spectrum of analytes. Additionally, 1-decanol remains a liquid at room temperature, whereas 1-dodecanol has a higher melting point and may solidify under typical laboratory conditions, making it more challenging to handle and apply.

#### Effect of extraction solvent volume

Volume of the extracting solvent used has a significant impact on the enrichment factor (EF), which quantifies the concentration of the analyte in the final extract. In general, using a smaller volume of extraction solvent increases the EF by concentrating the analyte in a small extract volume. However, if the volume is too small, phase separation may become difficult, hindering operational feasibility and overall extraction efficiency. On the other hand, increasing the solvent volume improves analyte recovery but also causes dilution, ultimately reducing the EF. Consequently, the precise selection of solvent volume plays an important role in attaining a high EF while ensuring effective phase separation and optimal analyte partitioning.

The optimization of 1-decanol volume was conducted by testing different amounts (30, 50, 75, 100, 125, and 150 µL) in triplicate (*n* = 3). However, LLME with 30 µL was challenging, as it was practically impossible to draw out the upper layer effectively after phase separation. From the tested volumes, 50 µL yielded the highest peak area, indicating superior extraction efficiency, and was thus selected for subsequent experiments. As shown in Fig. S1a, increasing the extraction solvent volume beyond 50 µL resulted in a gradual decrease in peak area, likely due to dilution effects, which reduced the enrichment factor.

#### Effect of extraction time

Optimizing extraction time is very important, as insufficient extraction durations may cause incomplete analyte partitioning, whereas excessively extended extraction times can lead to equilibrium conditions, beyond which further improvements in extraction efficiency become negligible. Extraction time is characterized as the period spanning from the introduction of the extraction solvent into the sample matrix to the onset of phase separation. Accordingly, extraction time was optimized by testing different manual shaking durations (0.5, 1.0, 1.5, 2.0, and 2.5 min), performed in triplicate (*n* = 3). Fig. S1b shows that a mixing time of 2 min provided the best results. This can be attributed to sufficient contact time between the extraction solvent and the sample matrix, allowing effective partitioning of analytes into the organic phase. Shorter durations likely did not provide adequate time for complete analyte transfer. Conversely, extending the mixing time beyond 2 min did not significantly enhance extraction efficiency, suggesting that equilibrium had already been reached. Therefore, a mixing time of 2 min was selected as the optimal extraction duration, balancing efficiency and practicality without exceeding the equilibrium point.

#### Effect of pH on the extraction efficiency

The pH of the sample solution significantly influences extraction efficiency through the alteration of the ionization state and solubility of the analytes. Suitable adjustment of pH can optimize the extraction of target compounds by favoring their neutral forms, which are more soluble in the organic extraction solvent, while ionized forms tend to remain in the aqueous phase. To assess the impact of pH on extraction efficiency, the water used for preparing the 25 µg mL^− 1^ BPA standard solution was adjusted at different pH values (4.0, 4.5, 5.0, 5.5 and 6.0) using 0.1 M HCl and 0.1 M NaOH, all in triplicate (*n* = 3). The pKa of BPA ranged from approximately 9.78 to 10.39, indicating that BPA predominantly existed in its neutral, molecular form at pH values below 9.78. Given that the investigated pH values are well below BPA’s pKa, BPA remained fully in its neutral form throughout the tested range. As a result, the extraction efficiency remained consistent, with no significant variation in the peak areas observed in the chromatographic analysis, as shown in Fig. S1c. This confirms that within this acidic to slightly acidic pH range, BPA’s ionization state, and thus its extractability remained unchanged. No pH adjustment was performed which not only simplified the procedure but also contributed to the method greenness.

#### Effect of salting-out on the extraction efficiency

The salting-out effect enhances the partitioning of analytes into the organic phase by reducing their solubility in the aqueous phase through salt addition. The concentration of the salt is a critical parameter, as it influences the extent of the salting-out effect as well as the efficiency of the extraction process. Therefore, its effect was studied by varying NaCl concentrations (0, 1, 2, 3, 4, and 5% w/v) in the water used to prepare the BPA working solution (25 µg mL^− 1^), all in triplicate (*n* = 3). As shown in Fig. S1d, at concentrations up to 4%, NaCl effectively reduced the solubility of BPA in water, resulting in improved extraction efficiency and a higher peak area. However, beyond 4%, the extraction efficiency declined. This decrease could be due to increased viscosity of the solution or saturation effects, which may hinder the mass transfer of BPA into the organic phase. Additionally, excessive salt can lead to phase separation issues or changes in solvent interactions, ultimately reducing the amount of BPA extracted.

**Fig. 2:**
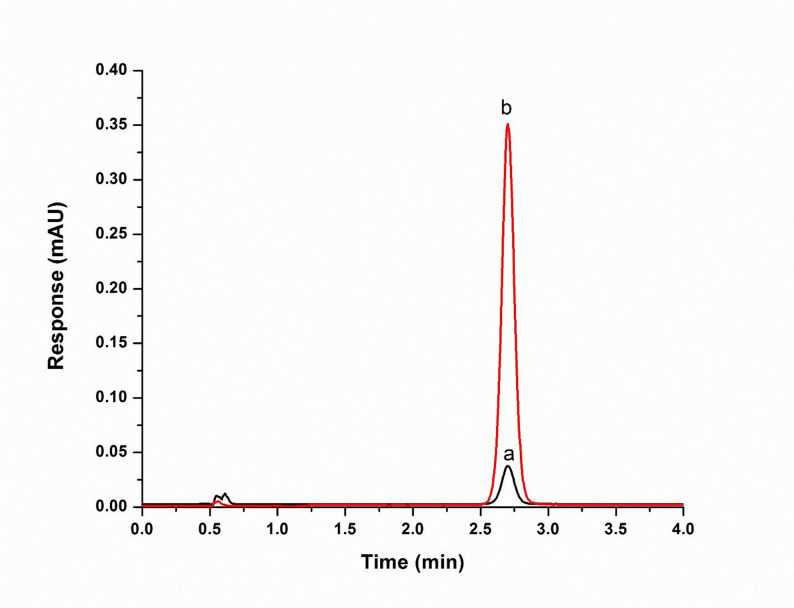
Chromatograms of bisphenol A before LLME (a) and after LLME (b). Chromatographic conditions: Column: XBridge^®^ C18 (4.6 mm × 50 mm i.d., 3.5 μm particle size); Mobile phase: ethanol–water (40:60, v/v); Flow rate: 1.00 mL/min; Detection: DAD at 277 nm; Injection volume: 10 µL

The optimal LLME conditions for efficient BPA extraction were established as follows: 1-decanol was selected as the extraction solvent due to its favorable physicochemical properties and environmental profile; a volume of 50 µL provided the highest enrichment without compromising phase separation; a mixing time of 2 min ensured sufficient contact for effective analyte partitioning; no pH adjustment was necessary as BPA remained in its unionized form across the tested range, and a NaCl concentration of 4% (w/v) enhanced extraction via the salting-out effect. As illustrated in Fig. 2, the chromatographic peak corresponding to BPA after applying the optimized LLME conditions was significantly enhanced compared to that obtained before optimization, confirming the improved extraction efficiency of the developed method.

### Method validation

The calibration curves (*n* = 8) for BPA were generated by plotting peak area against concentration (ng mL^− 1^). The linearity was assessed over the concentration range of 25–20,000 ng mL^− 1^, with correlation coefficients of 0.9989, demonstrating excellent linearity. Besides, analytical characteristics of the optimized method, including linearity, range, limits of detection (LODs), limits of quantification (LOQs) and regression parameters values were evaluated and listed in Table 1.

To ensure the reliability of the developed method, its validity was assessed in terms of accuracy and precision. Accuracy and precision were assessed in triplicate (*n* = 3). Accuracy was evaluated and the percent recovery values ranged from 99.60% to 100.85%, indicating minimal systematic errors and confirming the method’s accuracy.

**Table 1:** Combined Analytical Performance Data for the Determination of BPA using proposed method

		I. Regression Analysis			
Regression Parameters and Limits	Value	Unit	Parameter	Value	Unit
Linearity Range	25 − 20,000	ng mL^− 1^	**LOD** (Detection Limit)	8.33	ng mL^− 1^
Correlation Coefficient (*r*)	0.9989	(*n = 8*)	**LOQ** (Quantitation Limit)	25	ng mL^− 1^
Intercept (*a*)	0.07878	---	**Residual Std. Dev. (***Sy*/*x***)**	6.03 × 10^− 2^	(10^− 3^)
Slope (*b*)	0.22	(10^− 3^)	**Std. Error of Intercept (** *Sa* **)**	2.62 × 10^− 2^	(10^− 3^)
			**Std. Error of Slope (** *Sb* **)**	3.30 × 10^− 6^	(10^− 4^)

II. Accuracy and Precision Data (*n* = 3)						
Concentration Taken (ng mL^− 1^)	Accuracy and Intraday Precision			Interday Precision		
	Mean Conc. Found (ng mL^− 1^)	Recovery (%)	RSD (%)	Mean Conc. Found (ng mL^− 1^)	Recovery (%)	RSD (%)
25	24.95	99.80	2.28	24.98	99.60%	1.45
500	503.07	100.61	1.94	504.23	100.85%	1.15
5000	4996.82	99.94	0.45	5019.67	100.39%	0.65
10,000	9990.00	99.90	1.72	10019.96	100.79%	0.77

Precision was assessed in terms of intra-day (repeatability) and inter-day (intermediate precision) variations. Intra-day precision was determined by analyzing three replicates of the same sample within a single day, while inter-day precision was evaluated over three consecutive days. The relative standard deviation (RSD%) values were found to be ≤ 3.0% (except for the LLOQ), demonstrating excellent precision and minimal variability (Table 1).

### Real sample analysis

The developed method was applied by collecting 3 different water bottles and they were examined for BPA using the proposed LLME-HPLC/DAD approach. The findings revealed that BPA was not detected in any of the commercial water bottles. Each water sample was then spiked at three BPA concentration levels: low, medium and high (1 µg mL^− 1^, 5 µg mL^− 1^, 10 µg mL^− 1^), three replicates each. The recoveries ranged from 98.23% to 101.7%, with RSDs % as shown in Table 2. Our method proved to have the ability to detect trace amounts of BPA if found in any drinking water sample with remarked sensitivity and precision.

**Table 2:** The recovery data of BPA from different water samples were evaluated at three spiking levels, namely low (1 µg mL⁻¹), medium (5 µg mL⁻¹), and high (10 µg mL⁻¹), respectively

Samples	Contents (µg mL^− 1^) RSD%, *n* = 3	Spiked (µg mL^− 1^)	Average recovery (% ± RSD%, *n* = 3)
Dasani ®	Not detected	10	98.23% ± 1.04
	Not detected	5	99.32% ± 0.41
	Not detected	1	98.46% ± 0.95
Nestle ®	Not detected	10	101.73% ± 1.58
	Not detected	5	98.61% ± 2.09
	Not detected	1	98.94% ± 1.36
Eau ®	Not detected	10	98.32% ± 2.20
	Not detected	5	99.81% ± 0.42
	Not detected	1	101.11% ± 0.69

The developed LLME HPLC/DAD method was also applied to two different pharmaceutical products (Farcolin**®**, Tobrin®) and the two pharmaceutical samples were treated in the same way as the water bottles. The recoveries ranged from 98.60% to 99.22%, with RSDs % as shown in Table S2. These results indicate that the proposed method can also be applied to detect BPA leaching in pharmaceutical dosage forms packaged in plastic containers such as eye and ear drops.

## Greenness assessment for the proposed method

The concept of establishing green analytical chemistry (GAC) was to limit any environmental intrusion from analytical procedures. Our proposed method includes green characteristics such as a microextraction technique, green extracting and separation solvents, without any prior derivatization or toxic reagents, in alignment with the broader principles of sustainability and circular economy [38, 39]. All of these features contributed to the greenness of the proposed method. To assess the extent to which our method is green, the Analytical Eco-Scale [40], Modified Green Analytical Procedure Index (MoGAPI) [41], AGREE [34], AGREEprep [42] and the Environmental, Performance, and Practicality Index (EPPI) [35] were employed.

The Analytical Eco-Scale evaluates the greenness of an analytical procedure by assigning a numerical score. Since the score is determined by deducting penalty points (PPs) from the baseline value of 100. PPs are calculated based on factors such as the use of hazardous reagents, waste generation, occupational exposure risks, and high energy consumption [40]. A score of more than 75 represents an excellent green analysis, while a score above 50 indicates an acceptable green analysis, and a score below 50 is considered an inadequate green analysis. By applying this type of assessment for our method, the Analytical Eco-Scale score given was 88, indicating that the proposed method ensures excellent greenness as shown in Table 3.

**Table 3:**
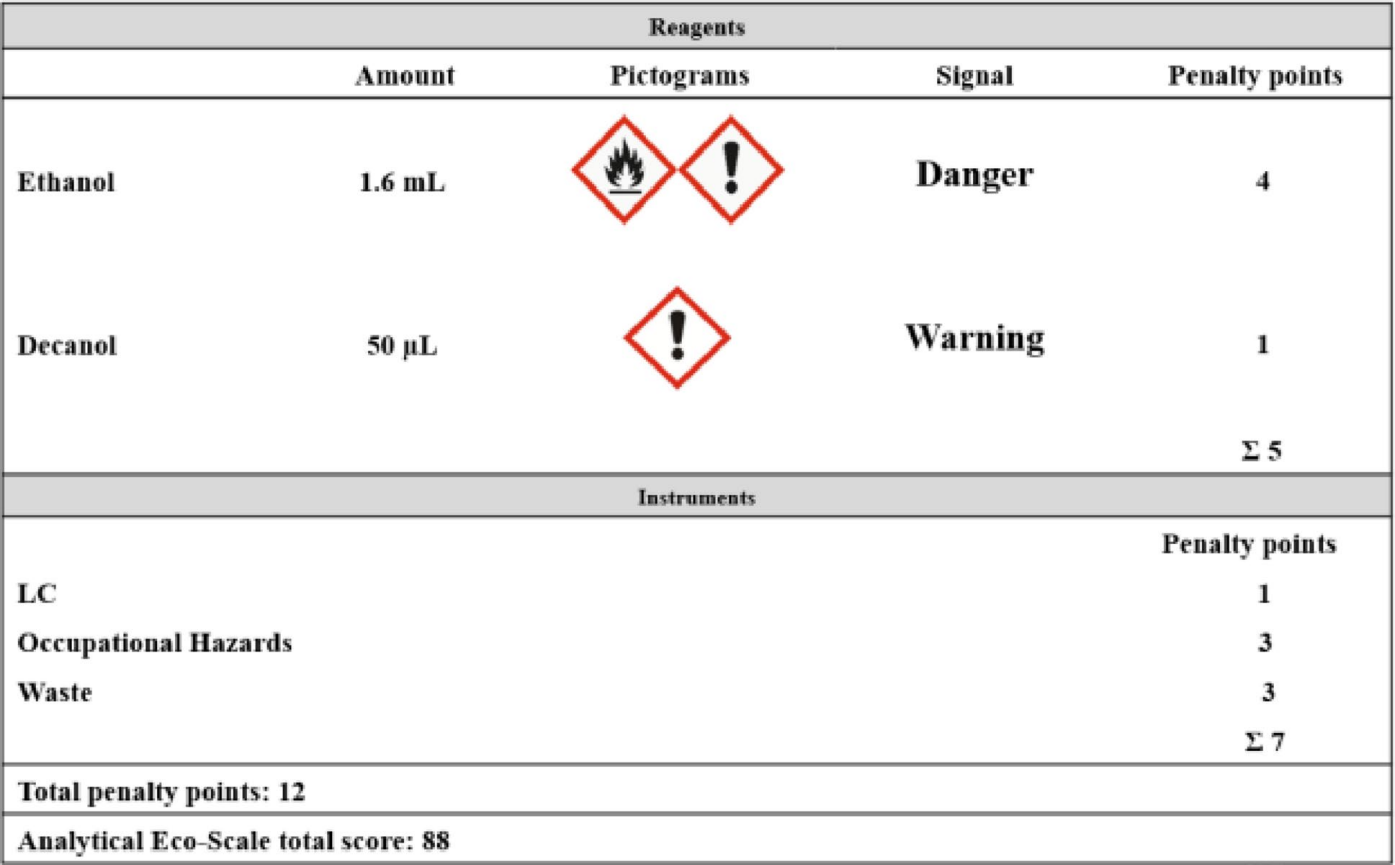
The penalty points (PPs) for BPA determination by the proposed LLME HPLC/DAD

**Fig. 3:**
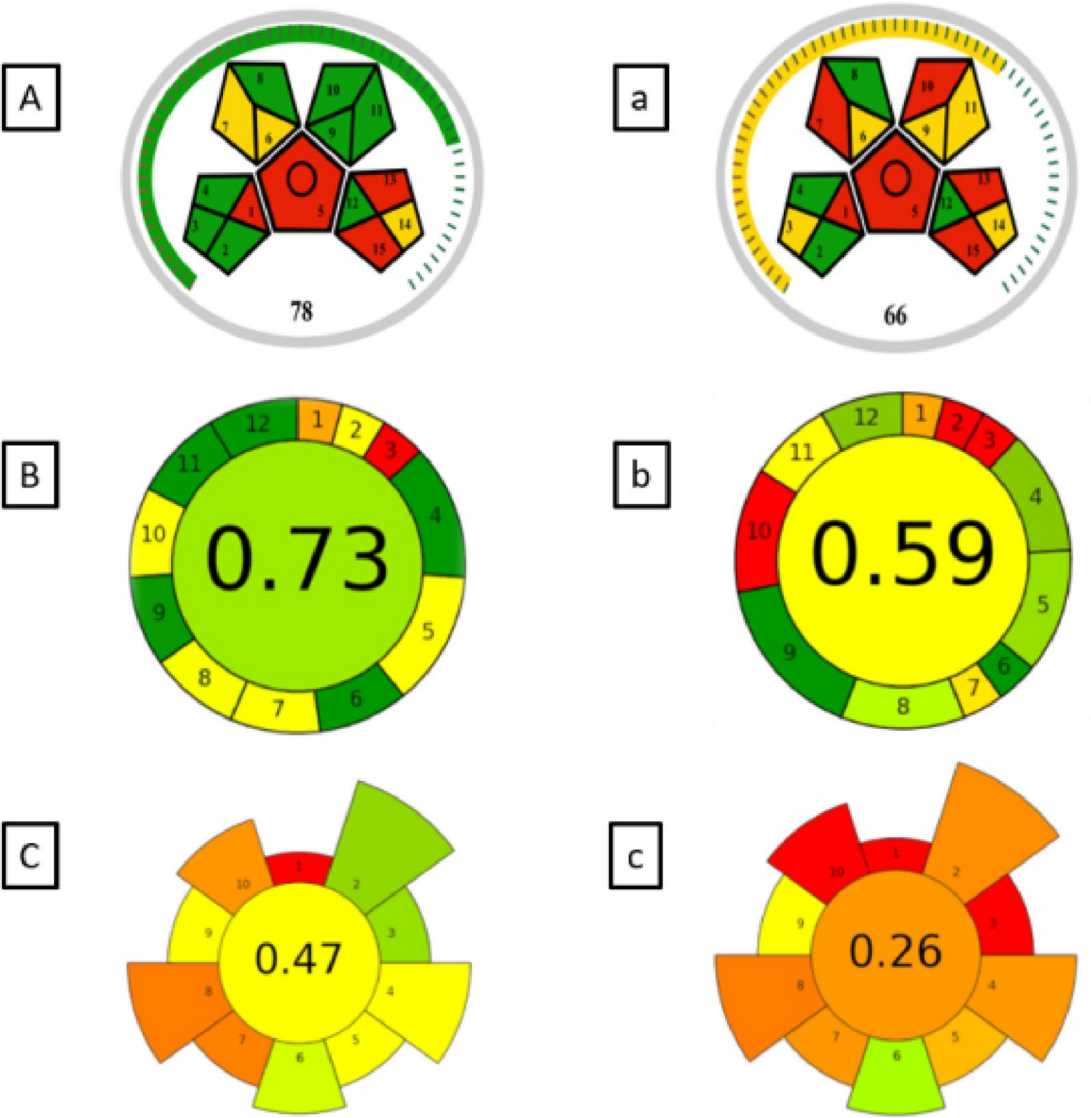
Greenness profile of the developed procedure (A, B, C) and the reported method (a, b, c) for determining BPA by LLME in water samples by MoGAPI (A, a), AGREE (B, b) and AGREEprep (C, c).

Among the various tools employed for green assessment, the Green Analytical Procedure Index (GAPI) is widely utilized to evaluate the overall sustainability of an analytical method. This assessment encompasses all procedural stages, from sampling and sample preparation to the final instrumental determination. GAPI employs a distinct graphical representation consisting of five pentagrams, which facilitate the interpretation and classification of environmental impact across different stages of the methodology. The evaluation follows a color-coded system, where green denotes minimal environmental impact, yellow signifies a moderate effect, and red represents a high impact [33, 43]. A modified version of the GAPI (MoGAPI) has been developed to integrate and combine the visual impact of GAPI with the quantitative accuracy of the Analytical Eco-Scale [41]. By applying this tool to our proposed method, a MoGAPI pictogram was generated, supporting the score stated above from the Analytical Eco-Scale as shown in Fig. 3A.

The AGREE framework was also adopted as an innovative assessment tool designed to incorporate each principle as a distinct input criterion. The effectiveness of the procedure in relation to each principle was represented using an intuitive color gradient, ranging from red to yellow to green, indicating varying degrees of environmental impact. In addition, the relative significance of each principle is visually conveyed through the proportional width of its corresponding segment [34]. AGREE metric system as shown in Fig. 3B was employed to measure the greenness of our analytical procedure.

While the previous methods evaluated analytical procedures, the sample preparation procedures are often underrepresented. For this reason, a new tool (AGREEprep) was developed including the 10 principles of green sample preparation (GSP) as assessment criteria [42]. AGREEprep was applied to our proposed method (as shown in Fig. 3C) with the results indicating superior greenness.

**Fig. 4:**
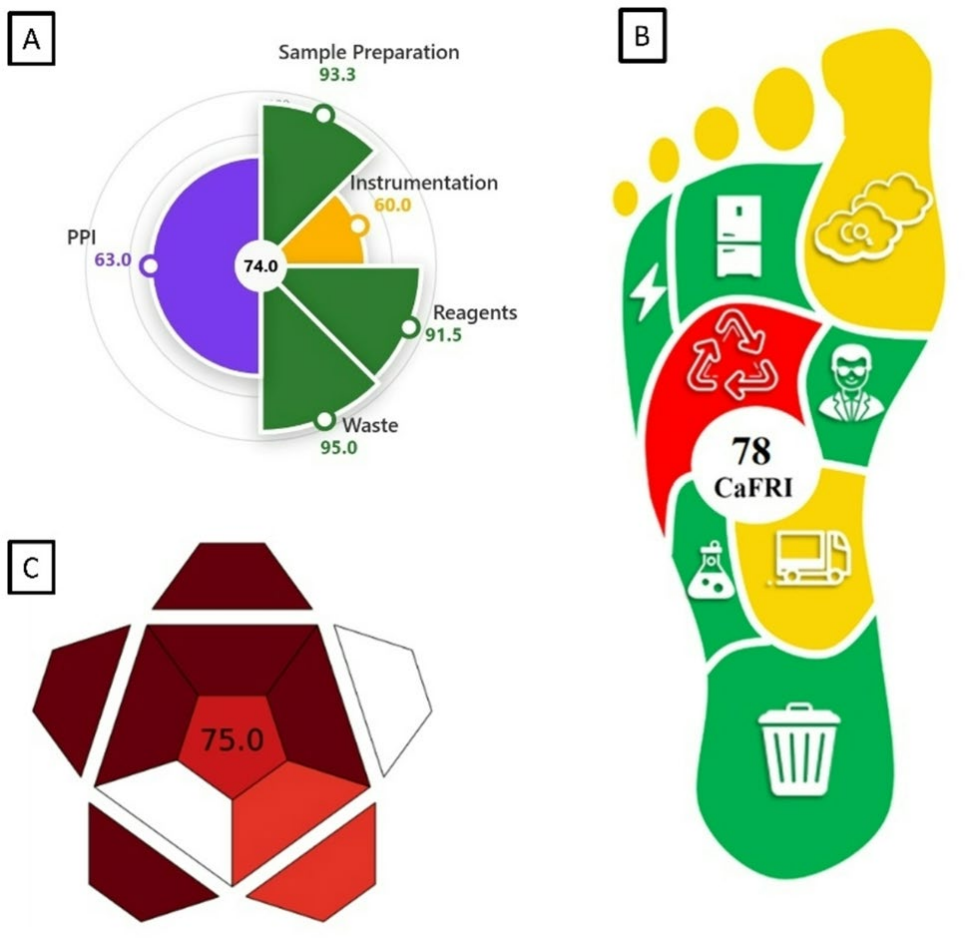
Environmental, Performance, and Practicality Index (EPPI) score (A), Carbon Footprint Reduction Index (CaFRI) score (B), and Red Analytical Performance Index (RAPI) score (C) of the proposed method.

To extend the hazard assessment beyond environmental burden and evaluate chemical safety more comprehensively, EPPI was also applied. EPPI assigns weighted hazard scores to each reagent based on toxicity, environmental persistence, bioaccumulation, and occupational risks. The EPPI evaluation of our method yielded an overall score of 74%, placing the method in the recommended category. Specifically, the Environmental Index (EI) reached 85%, reflecting an ideal green profile, while the Performance and Practicality Index (PPI) scored 63%, indicating acceptable practicality for routine laboratory application. These results are visually summarized in Fig. 4A, highlighting the favorable performance of our proposed method according to the EPPI metric. These results indicate the benign safety and environmental characteristics of ethanol and 1-decanol when compared with conventional solvents such as acetonitrile, chloroform, or methylene chloride. Integration of EPPI therefore reinforces the findings of the Eco-Scale, MoGAPI, AGREE, and AGREEprep assessments, collectively confirming the low hazard footprint and strong overall greenness of the proposed analytical procedure.

To fruther illsurtae the sustainability and performance of the developed LLME-HPLC/DAD method for BPA, the Carbon Footprint Reduction Index (CaFRI) [44] and the Red analytical procedure index (RAPI) [45] metrics were applied. As shown in Fig. 4B, the CaFRI score was 78, indicating a high level of environmental friendliness. The visual footprint highlights strong performance (green) in key areas such as waste generation, energy consumption, and chemical usage. Moderate performance (yellow) was noted in CO₂ emissions, transportation, and personnel safety, while the recycling-related parameter showed a lower score (red), suggesting room for improvement in solvent reusability. Meanwhile, the RAPI score, shown in Fig. 4C, was 75.0, reflecting a high degree of method performance. These results confirm that the proposed LLME-HPLC/DAD method is not only effective and sensitive but also sustainable and highly applicable for routine analysis.

**Fig. 5:**
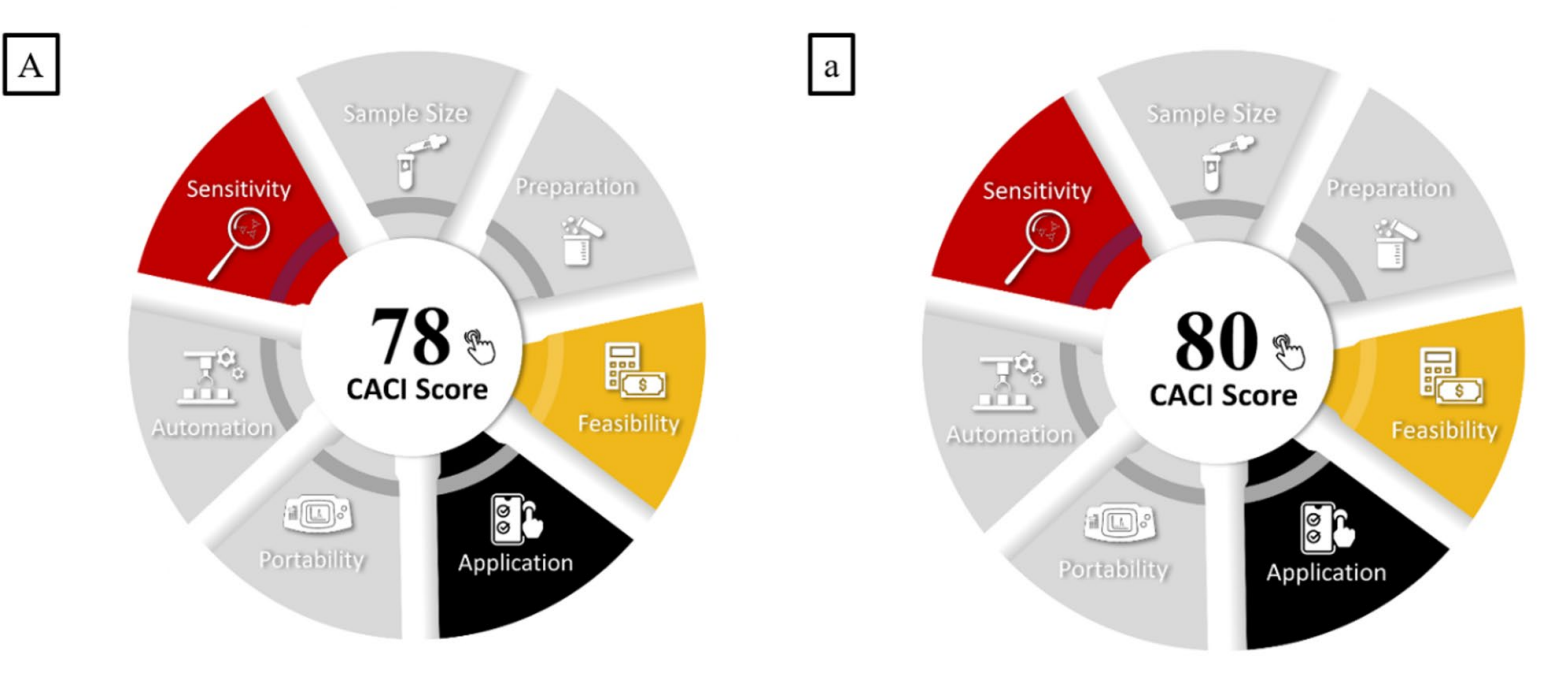
Assessment of the practicality of the developed procedure (A) and reported method procedure (a) for determining BPA by LLME in water samples by CACI

All the previously discussed metric systems do not fully consider for the practicality of analytical procedures. This aspect is crucial and should not be overlooked, as it directly impacts routine operations in analytical laboratories. Accordingly, the Click Analytical Chemistry Index (CACI) was presented as an innovative metric designed to assess the practical applicability of analytical techniques. This novel tool ensures that methodologies are not only theoretically efficient but also suitable for routine laboratory use and field-based applications. The color-coded pictogram in the CACI assessment system provides a visual representation of the method’s performance across various aspects. A colored pictogram signifies excellent performance, whereas a gray pictogram indicates moderate performance. In contrast, a black pictogram denotes inadequate performance or non-compliance with the established criteria [42]. As illustrated in Fig. 5A, the CACI evaluation confirms that our method demonstrates practical applicability.

### Comparison of our proposed method with other reported methods

The proposed method demonstrated superior environmental friendliness compared to the previously reported method [28], primarily due to the exclusion of non-green solvents such as chloroform, acetone, and acetonitrile. These solvents pose significant hazards; chloroform is a suspected carcinogen and hepatotoxin, acetone is highly flammable and can cause respiratory irritation, while acetonitrile is toxic and can lead to cyanide poisoning upon metabolism. Each of these solvents is associated with two hazard pictograms and the signal word “Danger,” resulting in a higher penalty score of 19 in the Analytical Eco-Scale assessment as shown in Table S3. In contrast, the proposed method achieved a lower penalty score of 12, indicating a more sustainable approach.

To provide greater clarity and transparency regarding the benchmark used for comparison, the detailed analytical parameters of the reported method have now been summarized in Table S4. Although this reported method is highly sensitive and incorporates a microextraction step, its dependence on hazardous organic solvents substantially compromises its greenness. This makes it a relevant and meaningful reference point, as it represents a method with strong analytical capabilities but significant environmental drawbacks.

Furthermore, evaluations using the AGREE, MoGAPI tools and AGREEprep for sample preparation confirmed that the proposed method achieved a higher greenness score than the reported method as shown in Fig. 3a, 3b and 3c. Practicality was assessed by employing CACI assessment tool (Fig. 5a). Our proposed methods achieved almost the same practicality level as the reference method, but it showed superior green characteristics as shown in the other green metrics.

## Conclusion

The proposed LLME-HPLC/DAD method provided a green, efficient, and sensitive approach for the detection of BPA in water samples. By optimizing critical extraction parameters and employing a low-toxicity solvent, this study demonstrates a practical alternative to conventional BPA detection methods. The method exhibited excellent linearity, low detection limits, and high precision, making it suitable for routine environmental monitoring. In addition, comprehensive greenness and performance assessments using Analytical Eco-Scale, MoGAPI, AGREE, AGREEprep, EPPI, CaFRI, RAPI and CACI confirmed its environmental sustainability and performance viability, achieving an excellent Analytical Eco-Scale score of 88 and strong performance in the green, blue and red metrics. This research highlights the importance of integrating eco-friendly microextraction techniques into analytical methodologies, paving the way for safer and more sustainable chemical analysis. The findings of this work open several promising avenues for further investigation. The method can be extended to a broader range of endocrine-disrupting chemicals or structurally related phenolic contaminants, enabling multi-residue screening. Its application can also be expanded to different water matrices, including surface water, groundwater, wastewater and industrial effluents, to assess matrix effects and ensure broader environmental applicability. Integration with portable or miniaturized HPLC systems may further support on-site or field-based water quality assessment. Additionally, coupling this extraction approach with mass spectrometric detection may enhance sensitivity and selectivity, aligning the method with regulatory-level monitoring requirements.

## Supplementary Information

Below is the link to the electronic supplementary material.

Supplementary Material 1

## Data Availability

The datasets used and/or analysed during the current study are available from the corresponding author on reasonable request.
