# Supplementary material for: Trio-colored appraisal of an eco-conscious method for the determination of bisphenol A in drinking water bottles and pharmaceutical eye-drop solutions
Source: BMC Chem. 2026 Jan 23;20(1):29. doi: 10.1186/s13065-025-01713-w (PMC12911365; doi:10.1186/s13065-025-01713-w)
Supplement: Supplementary file 1 — Supplementary Material 1 [file 13065_2025_1713_MOESM1_ESM.docx]

**Trio-Colored Appraisal of an Eco-Conscious Method for the Determination of Bisphenol A in Drinking Water Bottles and Pharmaceutical Eye-Drop Solutions**

**Supplementary materials**

**
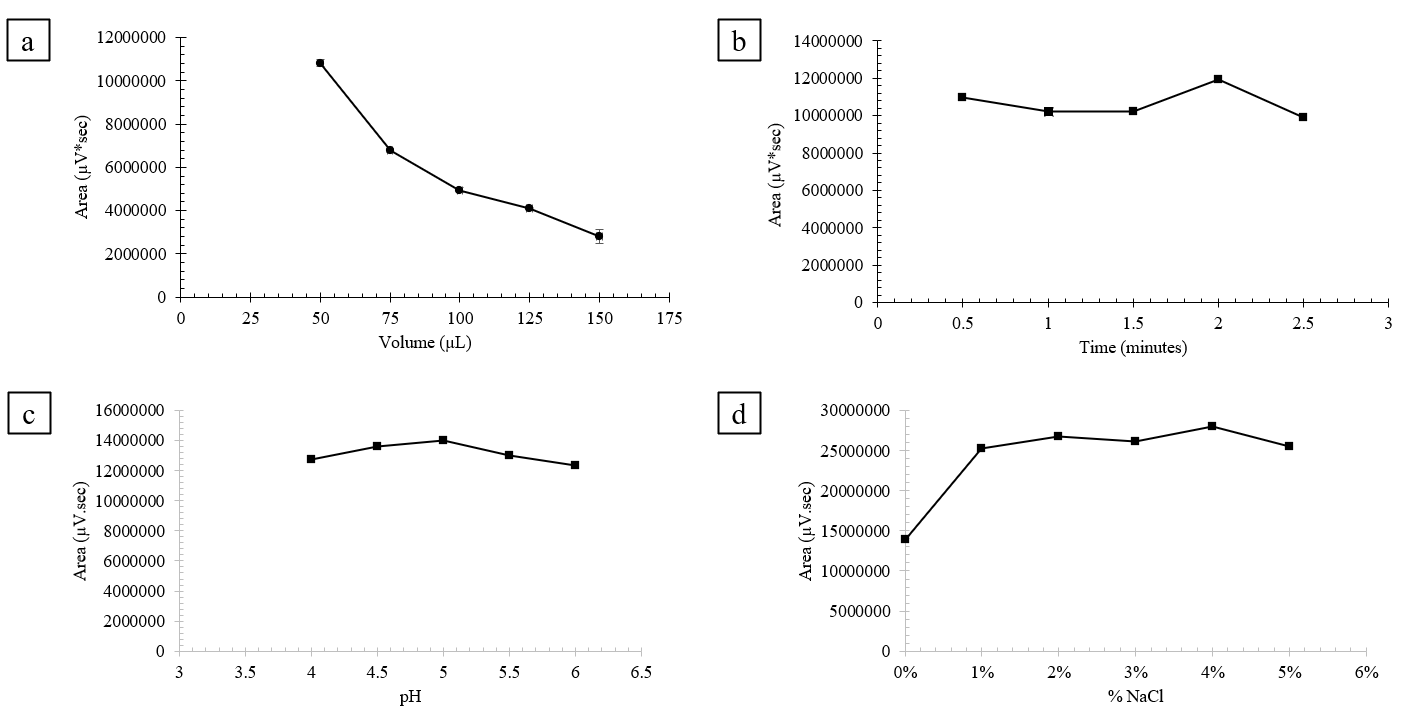
**

**Fig. S1.** Effect of extraction solvent volume (a), extraction time on extraction efficiency of BPA obtained from LLME (b), pH (c) and salting out using different concentration of NaCl (%w/v) (d).

**Table S1:** System suitability parameters of the proposed method for the determination of BPA.

| **Parameter** | **Result** | **Accepted limit** |
| --- | --- | --- |
| Retention time (min) | 2.83 | None |
| Retention Factor (k) | 5.1 | 2 -10 |
| Number of theoretical plates (N/column) | 2283 | 2000 – 10,000 |
| Tailing Factor | 1.36 | ≤ 2.0 |

**Table S2 :** The recovery data of BPA from different pharmaceutical products were evaluated at three spiking levels, namely low (1 µg mL⁻¹), medium (5 µg mL⁻¹), and high (10 µg mL⁻¹), respectively.

| **Samples** | **Contents**  **(μg/ mL) RSD%, n = 3** | **Spiked**  **(μg mL^-1^)** | **Average recovery**  **(% ± RSD%, n = 3)** |
| --- | --- | --- | --- |
| **Farcolin^®^** | Not detected | 10 | 98.93 % ± 0.76 |
|  | Not detected | 5 | 99.01 % ± 1.16 |
|  | Not detected | 1 | 98.60 % ± 0.82 |
| **Tobrin^®^** | Not detected | 10 | 98.68 % ± 1.32 |
|  | Not detected | 5 | 99.22 % ± 0.78 |
|  | Not detected | 1 | - 1. % ± 1.17 |


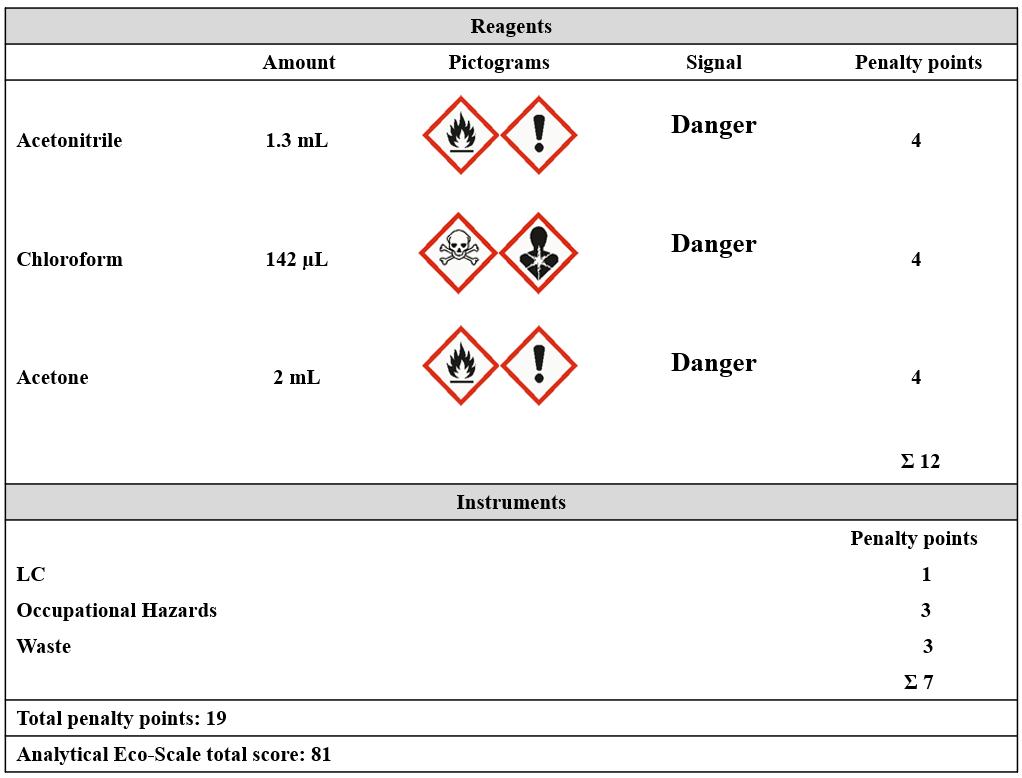
**Table S3:** The penalty points (PPs) for reported method.

**Table S4:** Detailed Parameters of the Reported DLLME–HPLC Method for BPA Determination.

| **Parameter** | **Value / Description** |
| --- | --- |
| 1. **Analytical Technique** | Dispersive Liquid–Liquid Microextraction (DLLME) coupled with HPLC–UV |
| 1. **Analyte** | Bisphenol A (BPA) |
| 1. **Calibration Range** | 0.5–100 µg/L |
| 1. **Calibration Curve (r²)** | 0.997 |
| 1. **LOD** | 0.07 µg/L (S/N = 3) |
| 1. **Precision (RSD%)** | 6.0% (n = 5, at 100 µg/L) |
| 1. **Accuracy / Relative Recovery** | 93.4–98.2% (tap water and river water) |
| 1. **Sample Volume** | 10.0 mL |
| 1. **Disperser Solvent** | Acetone, 2.0 mL |
| 1. **Extractant Solvent** | Chloroform, 142 µL |
| 1. **Injection Volume** | 20 µL |
| 1. **HPLC Column** | Zorbax Extend C18 (150 × 4.6 mm, 3 µm) |
| 1. **Mobile Phase** | Water : Acetonitrile (55:44, v/v), isocratic |
| 1. **Flow Rate** | 1.0 mL/min |
| 1. **Detection Wavelength** | 224 nm |
| 1. **Instrument Used** | Varian HPLC system (9012 pump, 9010 autosampler with 20 µL loop, 9050 UV–vis detector) |
| 1. **Replicates** | All experiments performed in triplicate |

**Graphical abstract**

**
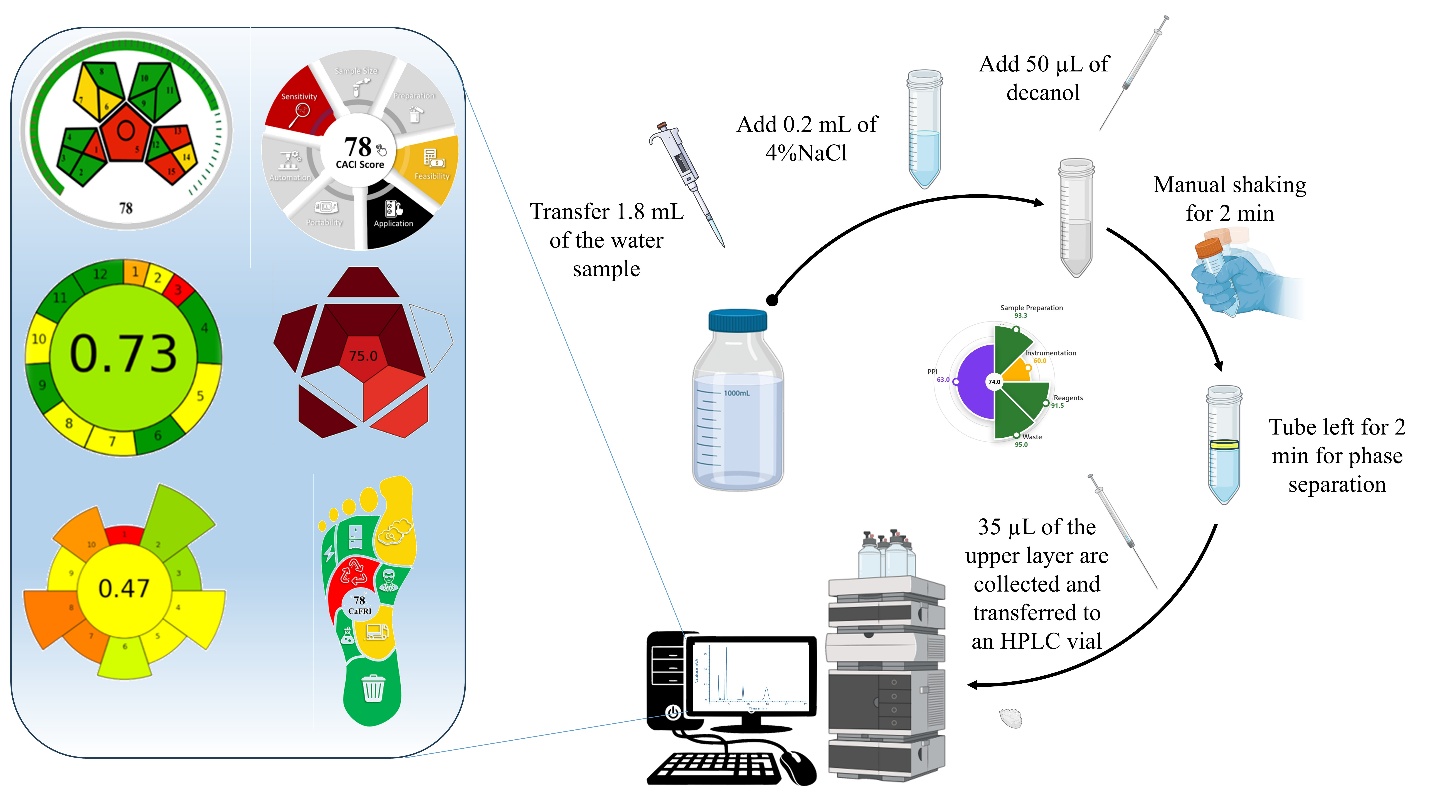
**
